# Localization of the Complement C1q-Binding Site on *Echinococcus multilocularis* Calreticulin Identified by Peptide Mapping

**DOI:** 10.3390/tropicalmed11060146

**Published:** 2026-05-26

**Authors:** Yinghui Song, Meng Xia, Haoran Zong, Wenjie Dong, Qiang Wang, Qin Yang, Bin Zhan, Yanhai Wang, Limei Zhao

**Affiliations:** 1 Department of Pathogenic Biology, School of Basic Medical Sciences and Forensic Medicine, Baotou Medical College, Baotou 014040, China; 13677153913@163.com (Y.S.); 18574858035@163.com (M.X.); lzzbodfn@163.com (H.Z.); 15849296910@163.com (W.D.); wqtwo@hotmail.com (Q.W.); 19834885733@163.com (Q.Y.); 2 Department of Pediatrics, National School of Tropical Medicine, Baylor College of Medicine, Houston, TX 77030, USA; bzhan@bcm.edu; 3 Parasitology Research Laboratory, School of Life Sciences, Xiamen University, Xiamen 361102, China

**Keywords:** *Echinococcus multilocularis*, calreticulin, C1q, binding site, immune evasion, neutrophil

## Abstract

Alveolar echinococcosis is a life-threatening zoonotic parasitic disease caused by infection of *Echinococcus multilocularis* larvae. To survive within the host’s immune milieu, *E. multilocularis* has evolved sophisticated immune evasion strategies, including the expression of immunomodulatory proteins that regulate the host immune response. Our previous studies have demonstrated that *E. multilocularis* calreticulin (*Em*CRT) possessed strong binding ability to human complement component C1q to inhibit C1q-initiated complement activation and biological functions. To further elucidate the mechanism by which *Em*CRT mediates C1q inactivation and immune evasion, the precise C1q-binding site on *Em*CRT was identified and analyzed in this study through expression of overlapping fragments and synthesis of overlapping peptides covering the identified functional fragment. The fragment expression and functional assay narrowed down the C1q-binding site to the *Em*CRT-S1 fragment located between amino acids 140 and 204 of *Em*CRT. The precise binding site was further pinpointed to the P5 peptide (*Em*CRT160–174 aa) by testing the synthetic peptides covering this region. The binding of peptide P5 to C1q markedly suppressed the activation of the C1q-mediated classical complement pathway and C1q-induced neutrophil chemotaxis, production of reactive oxygen species, cathepsin G, and myeloperoxidase. These findings suggest that the C1q-binding P5 peptide of *Em*CRT may serve as a potential target for the development of vaccines against echinococcosis or therapeutic drugs for complement-associated inflammatory or autoimmune diseases.

## 1. Introduction

As a severe zoonotic parasitic disease, echinococcosis presents two main types: alveolar echinococcosis (AE) and cystic echinococcosis (CE), which are caused by larval infections of *Echinococcus multilocularis* and *E. granulosus*, respectively [1]. Humans get infected by accidentally ingesting eggs released by infected animals—mainly dogs, foxes, and wolves—in their feces. AE is characterized by a tumor-like invasive growth, mainly involving the liver but possibly spreading to the lungs, brain, bones and other organs through the bloodstream or lymphatic system [2]. In the absence of prompt treatment, the fatality rate can be as high as over 90% [3]. AE is mainly distributed in the Northern Hemisphere, with reports in Europe, North America and Asia including China [4,5,6]. To establish persistent infection within the host, *E. multilocularis* has evolved complex immune evasion mechanisms [7,8]. Elucidating these mechanisms is critical for identifying vaccine and drug targets against *E. multilocularis* infection.

The complement system is an indispensable part of innate immunity, consisting of nearly 60 soluble and membrane-bound proteins [9]. It is activated through three pathways: the classical, the mannose-binding lectin, and the alternative pathway, all converging on C3 to form a membrane attack complex on the surface of invaded pathogens causing osmotic lysis and death [10]. As the initiating recognition molecule of the classical complement pathway, C1q not only initiates the complement cascade, but also exerts multiple non-complement-dependent immune functions. For instance, C1q induces the chemotaxis of macrophages, neutrophils and eosinophil to the infection site and enhances their ability to adhere to phagocytose and eliminate invaded pathogens. In particular, C1q interacts with its receptor on neutrophils to trigger the production of reactive oxygen species (ROS), cathepsin G and myeloperoxidase, which damage invaded pathogens or involve the development of autoimmune disease [11,12,13]. Notably, parasitic infestation can significantly alter peripheral blood counts [14] and is closely related to individual differences in specific and non-specific immunity. At present, most studies focus on immune changes under parasitic infection, while the underlying mechanism of host resistance to infection and parasite elimination remains poorly understood, which is modulated by genetic background, physiological conditions and potential immune specificity [15,16,17]. Therefore, further exploration of the regulatory role of C1q in anti-parasitic immunity is of important theoretical and practical significance.

Calreticulin (CRT) is a multifunctional protein comprising three distinct structural regions: a globular N-terminal domain, a proline-rich P domain, and an acidic C-terminal domain [18]. CRTs secreted by parasites play an important role in immunomodulating the host immune system especially by interfering with host complement functions by binding to human C1q as a survival strategy in the host [11]. Our previous research results demonstrated that *E. multilocularis*-expressed calreticulin (*Em*CRT) was able to interact with human C1q to suppress classical complement activation and C1q-induced mast cell migration [8], suggesting that *E. multilocularis* produces *Em*CRT to evade host complement-related attack. We further identified the C1q-binding region on *Em*CRT was localized to the S domain (*Em*CRT-S) spanning the N and P domains [19]. Nonetheless, the precise location of the C1q-binding site on *Em*CRT remains unclear. In this study, we expressed fragments within *Em*CRT-S to narrow down the C1q-binding region, then synthesized peptides that overlap the C1q-binding fragment with a goal to pinpoint the C1q-binding site at the amino acid level. The identification of the precise C1q-binding site will help us better understand the molecular interaction between C1q and *Em*CRT and the mechanism of *Em*CRT involved in the immune evasion. It also facilitates the design and production of the vaccine against echinococcosis or therapeutic drugs for complement-involved inflammatory or autoimmune diseases based on the identified C1q-binding amino acid peptide.

## 2. Materials and Methods

### 2.1. Serum

Normal human serum (NHS) was obtained from six healthy volunteers who had signed the informed consent form. This protocol has been reviewed and approved by the Institutional Review Board (IRB) of Baotou Medical College (Approval Number: 202510-8, 30 November 2025). The collected NHS was employed as the source of complement components. Human C1q-depleted serum (C1qD) was obtained from Quidel Corporation (San Diego, CA, USA).

### 2.2. Expression of Recombinant Protein Fragments

The C1q-binding domain of *Em*CRT has been located in the S-domain (*Em*CRT-S, 140–292 aa) [19]. To further locate the exact binding site of *Em*CRT to human complement C1q, the DNA sequences encoding for overlapped fragments of *Em*CRT-S (*Em*CRT-S1 140–204 aa, *Em*CRT-S2 184–248 aa, *Em*CRT-S3 228–292 aa) were obtained by PCR amplification from the total cDNA of *E. multilocularis*. The PCR products were cloned into the prokaryotic expression vector pET-28a (Novagen, Darmstadt, Germany) with *Bam*H I and *Xho* I restriction sites. Sequencing-verified recombinant plasmids were transformed into BL21 (DE3) competent cells (TIANGEN, Beijing, China). The recombinant proteins with His-tags at both N- and C-terminuses were induced for expression using 1 mM IPTG at 25 °C overnight and purified from the lysate supernatant using a Ni^2+^ affinity chromatography column (Beyotime Biotechnology, Shanghai, China). The protein purity was analyzed by SDS-PAGE and confirmed by Western blotting with anti-His antibody (BOSTER Biological Technology, Wuhan, China) and mouse anti-r*Em*CRT sera. The protein concentration was quantified using a BCA protein concentration assay kit (Solarbio, Beijing, China).

### 2.3. Peptide Synthesis

To ultimately determine the amino acid sequence of the C1q-binding site on *Em*CRT, 11 overlapping peptides covering *Em*CRT-S (P1-P11, corresponding to *Em*CRT140–154 aa, *Em*CRT145–159 aa, *Em*CRT150–164 aa, *Em*CRT155–169 aa, *Em*CRT160–174 aa, *Em*CRT165–179 aa, *Em*CRT170–184 aa, *Em*CRT175–189 aa, *Em*CRT180–194 aa, *Em*CRT185–199 aa, *Em*CRT190–204 aa) were synthesized using solid-phase peptide synthesis technology (Sangon Biotech, Shanghai, China). The synthetic peptides were purified by desalting and achieved a purity of 95% and were verified by mass spectrometry.

### 2.4. Binding Assay of rEmCRT-S Fragments to C1q

ELISA: To determine the binding ability of the different recombinant fragments of *Em*CRT-S (r*Em*CRT-S1, r*Em*CRT-S2 and r*Em*CRT-S3) to human C1q, 96-well microplates were coated with different amounts of C1q (0, 0.5, 1, 2, 4, 8, 12 µg/mL) (Abcam, Cambridge, UK) in 100 µL carbonate-coated buffer (0.1 M NaHCO_3_/Na_2_CO_3_, pH 9.6) overnight at 4 °C. The plates coated with an equal amount of bovine serum albumin (BSA) were used as controls. After being blocked with 200 µL of 3% BSA in PBS at 37 °C for 1 h, the wells of the plates were incubated with 100 µL of 0.5 µM r*Em*CRT-S1, r*Em*CRT-S2 and r*Em*CRT-S3 in protein-binding buffer (20 mM Tris-HCl, 1 mM CaCl_2_, 50 mM NaCl, pH 7.4) at 4 °C overnight. After being washed with 1 × PBST (1 × PBS + 0.05% Tween-20) three times, the recombinant fragments bound on the wells were recognized with a mouse anti-His tag antibody (1:3000, BOSTER Biological Technology, Wuhan, China) and an HRP-labeled goat anti-mouse IgG (1:5000, BOSTER Biological Technology, Wuhan, China). The TMB substrate solution (Beyotime Biotechnology, Shanghai, China) was added to initiate the chromogenic reaction, and the reaction was terminated using stop solution purchased from Beyotime Biotechnology (Shanghai, China). Absorbance at 450 nm was detected using an enzyme analyzer (Biotek Instrument Co., Ltd., Beijing, China). All samples were detected in triplicate, and BSA-coated wells served as a negative control to detect non-specific binding.

Far Western Blot: Human C1q and BSA (4 µg each) were separated by 12.5% polyacrylamide gel and then transferred onto PVDF membranes (Merck, Darmstadt, Germany). The membranes were blocked with 5% skimmed milk, then incubated with 4 µg of each r*Em*CRT fragment (r*Em*CRT-S1, r*Em*CRT-S2 and r*Em*CRT-S3) in 1 mL of protein-binding buffer at 4 °C overnight. The binding of the r*Em*CRT fragments to membrane-immobilized C1q was detected using a mouse anti-His monoclonal antibody (1:5000) and an HRP-conjugated goat anti-mouse IgG (1:10,000), followed by visualization using a chemiluminescence imaging system (Tanon, Shanghai, China).

### 2.5. Dot Blot

To determine the binding ability of the synthetic peptide to C1q, each peptide and BSA (7 μg in 2 μL volume) were spotted onto a nitrocellulose membrane (LABSELECT, Beijing, China). After being blocked with 5% skimmed milk in PBS, the membrane was incubated with 3 μg/mL human C1q in the protein-binding buffer overnight at 4 °C and then probed with an anti-C1q antibody (1:1000, BOSTER Biological Technology, Wuhan, China).

### 2.6. Evaluation of Peptide P5-Mediated Suppression of Complement C4b and C3b Deposition

To evaluate the effect of peptide P5 on C1q-mediated complement activation, human IgM (2 µg/mL, Sigma, St. Louis, MO, USA) was coated onto a 96-well plate at 4 °C overnight, then blocked with 3% BSA at 37 °C for 2 h to prevent non-specific binding. To each well, 1 µg of C1q pre-incubated with different amount of P5 peptide (0, 2, 3 µg), r*Em*CRT (3 µg), or BSA (3 µg) in a total volume of 100 µL was added for incubation at 37 °C for 2 h. After thoroughly washing with PBST, 100 µL of C1q-D serum diluted 1:75 in GVBS++ buffer (1 × Veronal buffer containing 0.05% Tween-20, Lonza, Basel, Switzerland) was added to each well as a supplementary component other than C1q and incubated for 1 h at 37 °C to initiate the complement activation cascade. NHS (1:75) was used as a positive control. The cascade intermediators C4b and C3b were detected by a goat polyclonal antibody against human C4b (1:3000, Abcam, Cambridge, UK) or a rabbit monoclonal antibody against human C3b (1:1000, BOSTER Biotechnology, Wuhan, China) for 1 h at 37 °C, respectively. The HRP-conjugated rabbit anti-goat IgG (1:5000, Affinity Biosciences, Liyang, China) or the goat anti-rabbit IgG (1:1000, Affinity Biosciences Company, Liyang, China) were added as secondary antibodies with TMB used as the substrate. The absorbance was measured with a microplate reader at a wavelength of 450 nm.

### 2.7. Hemolysis Experiment

To evaluate the inhibitory effect of the P5 peptide on C1q-associated hemolysis mediated by the classical complement pathway, fresh sheep red blood cells (SRBCs, 5 × 10^8^ cells/mL) were incubated with rabbit anti-SRBC antibody (Zhengzhou Baiji Biotechnology Co., Ltd., Zhengzhou, China) in HBSS++ buffer (Hank’s Balanced Salt Solution supplemented 0.15 mM calcium chloride and 1 mM magnesium chloride, Solarbio, Beijing, China) at 37 °C for 30 min to achieve sensitization. One µg of C1q was pre-incubated with different amounts of P5 peptide (0, 1, 1.5, and 2 µg), r*Em*CRT (2 µg) and BSA (2 µg) at 4 °C overnight, then human C1qD serum diluted at 1:100 in HBSS++ was added into the mixture for 1 h at 37 °C to reconstitute complement activity. The sensitized SRBCs were then added into the reaction mixture for one more hour. The hemolysis reaction was terminated with cold HBSS++ containing 10 mM EDTA. After centrifugation at 1500× *g* for 10 min, the absorbance at 412 nm in the supernatant was determined. The hemolytic percentage was calculated relative to the total hemolysis in water.

### 2.8. HL60 Cells Culture and Differentiation

HL60, a human promyelocytic cell line, possesses multiple neutrophil receptors and can be induced to differentiate into neutrophil-like cells, rendering it suitable for functional studies of neutrophils. The HL60 cell line was purchased from Procell Life Sciences Co., Ltd. (Wuhan, China) and cultured in IMDM-specific medium (Procell system, Wuhan, China) supplemented with 20% fetal bovine serum (FBS, Procell system, Wuhan, China) and 1× penicillin-streptomycin. HL60 cells were treated with 1 µM all-trans retinoic acid (ATRA, Merck, Darmstadt, Germany) for 5 days to induce differentiation into neutrophil-like cells (dHL60) [20] with CD11b^+^ and adherent ability.

### 2.9. EmCRT-P5 Suppresses C1q-Stimulated dHL60 Cell Activity

To evaluate whether the short peptide P5 inhibits C1q binding to its receptors on the surface of neutrophil cells, dHL60 cells were seeded onto poly-L-lysine coated slides in a 24-well plate (5 × 10^4^ cells/well) and fixed with 4% polyformaldehyde. The fixed dHL60 cells on the slides were blocked at room temperature for 30 min with normal goat serum (ZSGB Biotechnology Company, Beijing, China). C1q (15 nM) pre-incubated with different concentrations of P5 (0, 20, 40 µg/mL) or r*Em*CRT (40 µg/mL) was transferred to the dHL60 cells on the slides. After washing, the cells were incubated with rabbit anti-C1q monoclonal antibody (1:200, BOSTER Biological Technology, Wuhan, China) overnight at 4 °C to measure the binding of C1q to dHL60 cells. DyLight 488-labeled goat anti-rabbit IgG (1:200) (BOSTER Biological Technology, Wuhan, China) was added as a secondary antibody. The nuclei were stained with DAPI (Beyotime Biotechnology, Shanghai, China). The fluorescent mages were collected using a confocal laser scanning microscope (Nikon, Tokyo, Japan).

### 2.10. Transwell Migration Assay

The inhibitory effect of peptide P5 on the C1q-stimulated chemotaxis of dHL60 cells was determined using 3 µm pore Transwell inserts (Nest Biotechnology, Wuxi, China). dHL60 cells were seeded into the upper chambers at 2 × 10^5^ cells/well in 200 µL of IMDM medium. C1q (20 nM) pre-incubated with different concentrations of P5 (8 and 16 µg/mL) or r*Em*CRT (16 µg/mL) in 500 µL IMDM medium was added to the lower chamber. The change in the chemotactic migration was observed 8 h after incubation at 37 °C with 5% CO_2_. The cells that passed across the membrane and migrated into the lower chamber were counted using a cell counter (Guangzhou Newton Optics Research Institute, Guangzhou, China). BSA (16 µg/mL) was used as a negative control.

### 2.11. Detection of Reactive Oxygen Species in dHL60

To evaluate the impact of P5 on ROS (reactive oxygen species) production in C1q-stimulated neutrophils, the fluorescent probe DCFH-DA (Biyun Tian Biotechnology, Shanghai, China) was used. A 96-well plate was coated with C1q (1 µg/well) and incubated with different amounts of P5 (4, 8 µg) or r*Em*CRT (8 µg) at 4 °C for 12 h. BSA (8 µg) and PMA (160 nM) served as the negative control protein and positive control, respectively. Then, 5 × 10^4^ dHL60 cells were seeded into each well and cultured overnight at 37 °C in a 5% CO_2_ incubator. ROS production in dHL60 cells was measured with 10 µM DCFH-DA, and the fluorescence intensity was measured using a fluorescence microplate reader (Thermo Fisher, Waltham, MA, USA).

### 2.12. Detection of Cathepsin G and Myeloperoxidase Activity in dHL60

As described above, dHL60 cells were stimulated with C1q that had been pre-incubated with different concentrations of P5 (3, 4 µg) or r*Em*CRT (4 µg). The levels of cathepsin G and myeloperoxidase (MPO) activity in dHL60 were then determined using commercial ELISA kits (Jiangsu MEIMIAN Industrial Co., Ltd.). The absorbance of each well was measured at 450 nm using an enzyme reader (Thermo Fisher, Waltham, MA, USA).

### 2.13. Statistical Analysis

The statistical analysis data were presented as mean ± standard error of the mean (SEM). The one-way analysis of variance was performed for comparisons among multiple groups using GraphPad Prism 10 software (San Diego, CA, USA). In all statistical analyses, *p* < 0.05 was considered statistically significant.

## 3. Results

### 3.1. Expression and Characterization of Recombinant EmCRT-S Fragments

**Figure 1:**
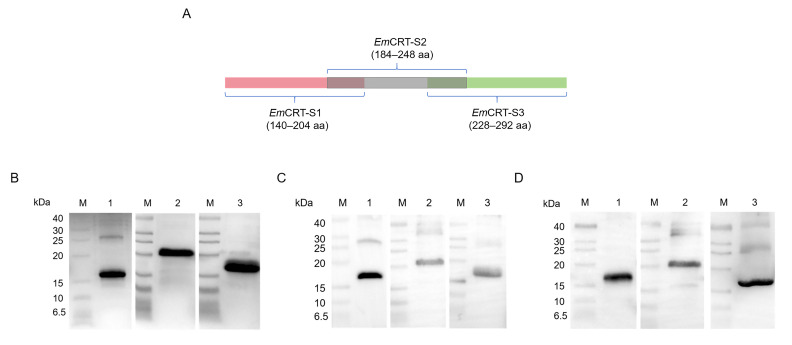
Characterization of expressed recombinant fragments of *Em*CRT-S. (**A**) Schematic diagram of overlapped *Em*CRT-S fragments. (**B**) The purified r*Em*CRT-S truncated fragments were analyzed by SDS-PAGE. (**C**) Western blot detection of 1 µg r*Em*CRT-S truncated fragments using mouse anti-His monoclonal antibody (**D**) and mouse anti-r*Em*CRT serum. (M, molecular weight marker; Lane1: r*Em*CRT-S1; Lane2: r*Em*CRT-S2; Lane3: r*Em*CRT-S3.)

Our previous results showed that the C1q-binding domain of *Em*CRT was located within the S domain (*Em*CRT-S, 140–292 aa) [19]. To determine the precise human C1q-binding region on *Em*CRT, three overlapped fragments within *Em*CRT-S including *Em*CRT-S1 (140–204 aa), *Em*CRT-S2 (184–248 aa) and r*Em*CRT-S3 (228–292 aa) were expressed in *E. coli* BL21 (DE3) and purified by nickel affinity chromatography (Figure 1A). The SDS-PAGE analysis revealed that the recombinant protein fragments displayed the molecular weight between 17 and 22 kDa (including N- and C-terminal His-tags), which is higher than the predicted size (≈12 kDa) based on their amino acid sequences, possibly due to the intermolecular reaction (Figure 1B) [21,22,23,24,25]. The purified recombinant protein fragments were strongly recognized by both the anti-His antibody (Figure 1C) and the anti-*Em*CRT immune serum (Figure 1D).

### 3.2. Localization of the C1q-Binding Fragment of EmCRT-S

**Figure 2:**
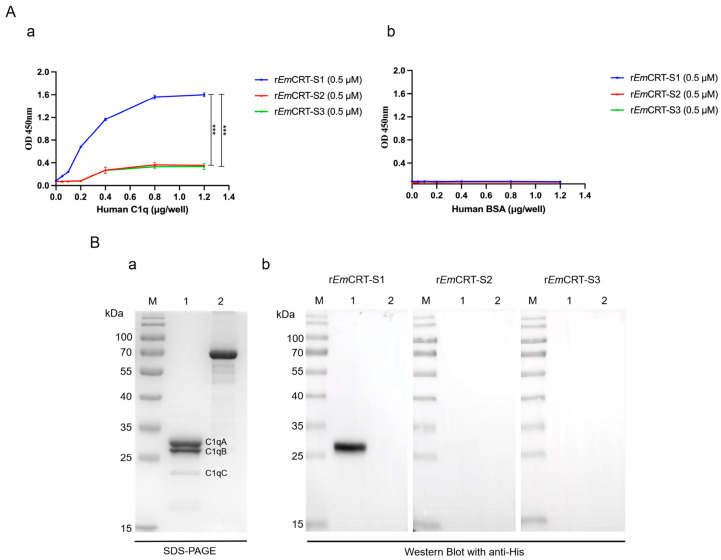
Binding capabilities of different fragments of *Em*CRT-S to human C1q. (**A**) Binding abilities of different r*Em*CRT-S truncated fragments to human C1q (0, 0.5, 1, 2, 4, 8, and 12 µg/mL) coated on 96-well plates were detected by ELISA using anti-His antibody. (**a**) No significant binding was observed with BSA at the same concentration gradient. (**b**) The experiment was performed in triplicate, and data are presented as the mean ± standard deviation (*** *p* < 0.001 compared to the plate with the addition of r*Em*CRT-S2 or r*Em*CRT-S3). (**B**) Far Western blotting was used to determine the binding capacity of different fragments of *Em*CRT-S to C1q transferred on PVDF membranes. A total of 4 µg of C1q and BSA were separated by SDS-PAGE under denaturing conditions. (**a**) For Far Western blotting analysis 4 µg of human C1q was transferred to a PVDF membrane and incubated with 4 µg/mL truncated fragments of *Em*CRT-S. (**b**) The bound recombinant fragment was detected using anti-His antibody (1:5000).

To further identify the binding capabilities of differently expressed fragments of *Em*CRT-S to human C1q, ELISA and Far Western blotting were performed. The ELISA results exhibited that only the r*Em*CRT-S1 fragment bound to C1q in a dose-dependent manner and reached saturation at 0.5 μM when incubating with 0.8 μg/well-coated C1q, whereas the other fragments showed no obvious binding to C1q. There was no binding of any fragment to the BSA control (Figure 2A). Far Western blotting demonstrated that r*Em*CRT-S1 exhibited the strongest binding to C1q, particularly to the A chain, with no detectable binding to BSA (Figure 2B), verifying the specificity of the interaction. Collectively, the C1q-binding region of r*Em*CRT was preliminarily localized within the *Em*CRT-S1 region.

### 3.3. Mapping of the C1q-Binding Peptide

**Figure 3:**
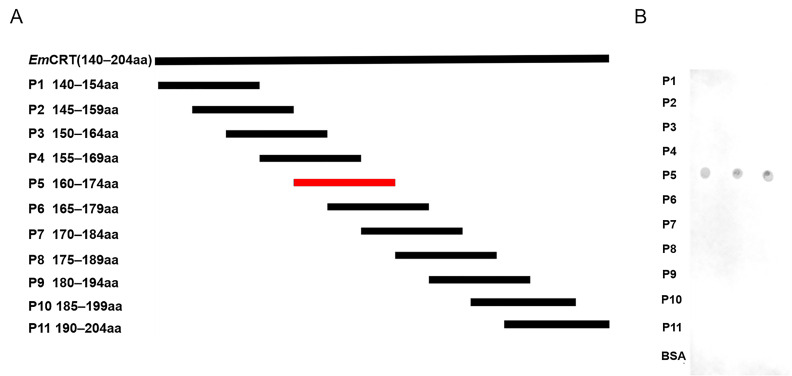
Mapping of the C1q-binding site on *Em*CRT-S using overlapping synthetic peptides. (**A**) Schematic representation of overlapping short peptides P1 to P11 covering the fragment *Em*CRT-S1. (**B**) Dot-blot analysis of synthetic peptides (7 µg in 2 µL) spotted onto nitrocellulose membranes. The same amount of BSA was spotted on the membrane as control. After blocking, the membrane was incubated with 3 µg/mL human C1q and detected using anti-C1qA monoclonal antibody (1:1000 dilution). Black lines indicate peptides without binding activity, the red line marks the binding-positive peptide P5.

To further pinpoint the C1q-binding site on r*Em*CRT, we synthesized 11 overlapping short peptides (P1-P11, 15 amino acids each) within the *Em*CRT-S1 fragment (Figure 3A) based on the results above showing that the C1q-binding region is localized in this fragment. The binding abilities of these peptides to human C1q were determined by dot blotting. The synthesized peptides were spotted onto nitrocellulose membranes, incubated with human C1q, and detected by a colorimetric assay using an anti-C1qA monoclonal antibody. The results showed that among the 11 short peptides tested, only peptide P5 exhibited specific binding to C1q, whereas other peptides and the irrelevant BSA control displayed no obvious binding signal (Figure 3B). These results determine the C1q-binding site located within the 15-amino-acid sequence corresponds to peptide P5 in *Em*CRT-S.

### 3.4. Suppression of Classical Complement Activation and Mediated Hemolysis by Peptide P5

**Figure 4:**
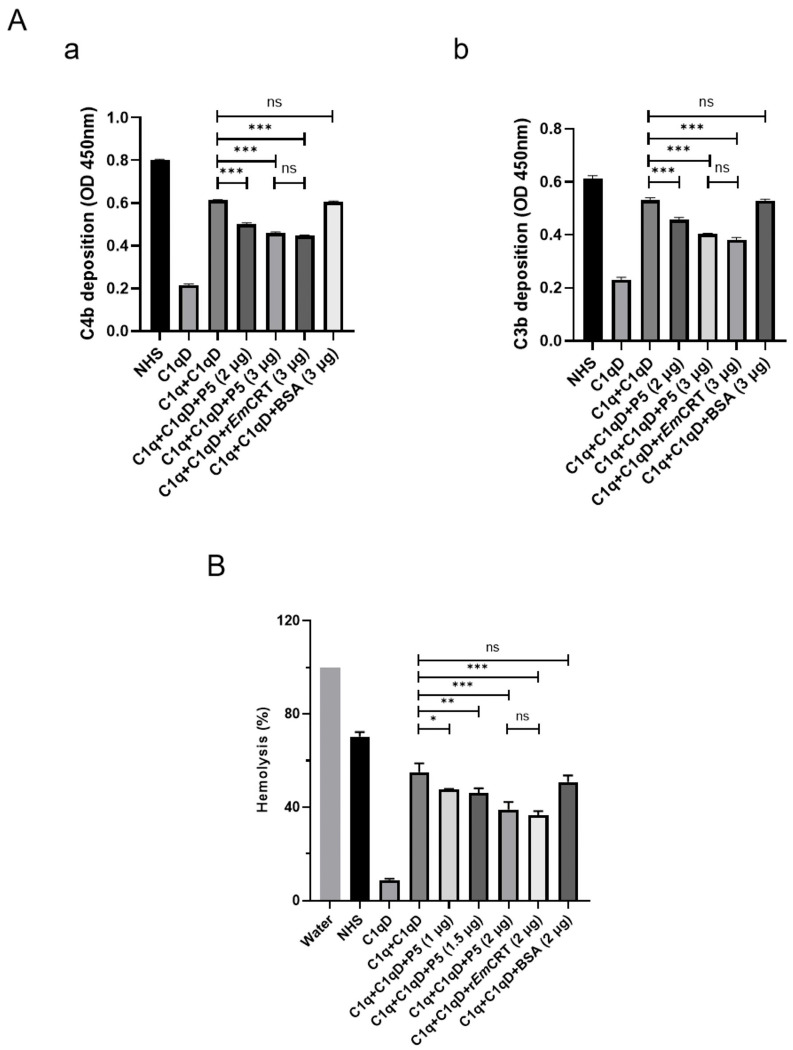
Inhibition of classical complement activation and the complement activation-mediated hemolysis by peptide P5. (**A**) The inhibitory effect of P5 on the deposition of C4b/C3b was analyzed by ELISA. C1q (1 µg/well) was pre-incubated with P5 (0, 2, 3 µg), r*Em*CRT (3 µg), or BSA (3 µg), and then added to 96-well plates pre-coated with human IgM (2 µg/mL). C1q-deficient serum was introduced to activate the IgM-C1q-driven classical complement pathway. The levels of deposited C4b (**a**) and C3b (**b**) were measured using anti-C4b (1:3000) and anti-C3b (1:1000) antibodies. (**B**) The inhibitory effect of P5 on the complement-mediated hemolysis was performed by measuring the OD412 in the supernatant. C1q was pre-incubated with P5 (1, 1.5, 2 µg), r*Em*CRT (2 µg), or BSA (2 µg), followed by the addition of C1q-D serum and sensitized sheep red blood cells (SRBC). BSA, C1q-D, and NHS alone were used as controls. Data are shown as mean ± SD of three independent experiments. (* *p* < 0.05, ** *p* < 0.01, *** *p* < 0.001, ns, no significant difference.)

To evaluate the inhibitory effect of peptide P5 on C1q-initiated complement activation, ELISA was performed to detect deposition of the C1q/IgM-initiated complement activation intermediator C3b/C4b on the plate. The results showed that pre-incubation of human C1q with peptide P5 significantly reduced the deposition of C3b and C4b in a dose-dependent manner (Figure 4A). The inhibitory level is similar to that of full-length *Em*CRT. A similar result was achieved from the inhibitory effect of P5 on the C1q-initiated hemolysis. The addition of C1q and C1qD serum to the sensitized sheep red blood cells resulted in approximately 60% hemolysis, whereas pre-incubation of C1q with P5 markedly suppressed hemolysis in a dose-dependent manner, comparable to the same amount of r*Em*CRT (Figure 4B). BSA had no effect on the hemolysis in the control group. These results verify that P5 inhibits the C1q-initiated classical complement activation pathway.

### 3.5. Inhibition of the C1q Binding to Neutrophils by Peptide P5

**Figure 5:**
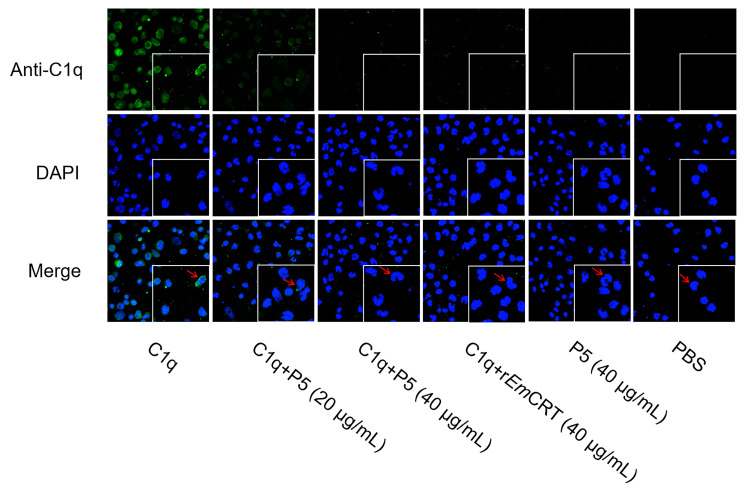
Confocal microscopy showing P5 inhibits C1q binding to dHL60 cells. dHL60 cells were seeded on glass slides. C1q pre-incubated with P5 (0, 20, 40 µg/mL) or 40 µg/mL r*Em*CRT was added to the dHL60 cells seeded on glass slides. Red arrows indicate the C1q bound to the cell surface. C1q binding to cells was detected with anti-C1q antibody and FITC-labeled goat anti-rabbit IgG (green); nuclei were stained with DAPI (blue). Magnification: 200×; magnification of the lower right image: 400×.

To investigate whether peptide P5 inhibits the binding of C1q to its receptor on neutrophil cells, HL60 cells were differentiated into neutrophil-like cells (dHL60) after 5 days incubation with 1 µM ATRA. Successful dHL60 differentiation was reflected by the elevated CD11b expression and characteristic neutrophil nuclear morphology observed by DAPI staining. C1q was pre-incubated with different concentrations of P5 (0, 20, 40 µg/mL) or 40 µg/mL r*Em*CRT and then incubated with dHL60 cells. The C1q binding on the neutrophils was detected by immunofluorescence assay using an anti-C1q antibody. The results showed that P5 inhibited the binding of C1q to dHL60 cells in a dose-dependent manner (Figure 5). No significant fluorescence was detected in cells treated with P5 or PBS alone without C1q. These results suggest that peptide P5 competitively blocks the interaction between C1q and its receptor on the surface of dHL60 cells by binding to C1q.

### 3.6. Inhibition of C1q-Induced Neutrophil Functions by Peptide P5

**Figure 6:**
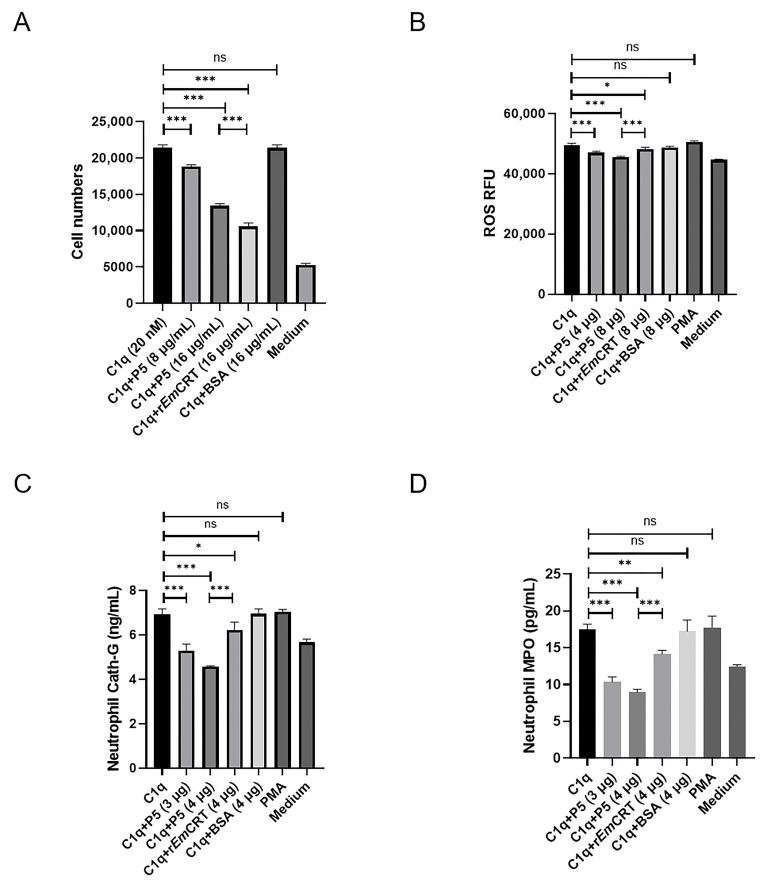
P5 inhibits C1q-induced neutrophil chemotaxis and the production of effectors. (**A**) Transwell migration assay showing that P5 suppressed C1q-induced chemotaxis of dHL60 cells. (**B**) ROS production was measured using DCFH-DA. (**C**) Neutrophil cathepsin G (Cath-G) production and (**D**) neutrophil myeloperoxidase (MPO) production measured using commercial ELISA kits in the cell supernatant. Data are representative of three independent experiments and expressed as mean ± SD. (* *p* < 0.05, ** *p* < 0.01, *** *p* < 0.001, ns, no significant difference.)

Transwell chemotaxis assays were performed to assess the effect of P5 on C1q-induced migration in dHL60 cells. The results showed that C1q alone significantly induced dHL60 cell transmembrane migration, whereas pre-incubation with P5 dose-dependently suppressed this chemotactic migration; however, the inhibitory effect is less than the same amount of full-length r*Em*CRT (Figure 6A). BSA showed no inhibitory effect. We further evaluated the effect of P5 on C1q-induced production of effector molecules in dHL60 cells, including reactive oxygen species (ROS), cathepsin G (Cath-G), and myeloperoxidase (MPO). C1q alone markedly elevated ROS production and secretion of Cath-G and MPO, which are critical for neutrophil anti-pathogen defense. Pre-incubation of C1q with P5 significantly reduced the production of C1q-induced neutrophil-related mediators ROS, Cath-G and MPO in a dose-dependent manner (Figure 6B–D). The inhibitory effect of P5 on the production of these mediators is significantly higher than that induced by the same amount of full-length r*Em*CRT. BSA did not affect C1q-induced responses, and PMA effectively stimulated the production of all indicators as a positive control. Collectively, these results demonstrate that the P5 peptide has a comparable effect to the full-length r*Em*CRT in negatively regulating C1q-mediated neutrophil activation and effector responses.

## 4. Discussion

Helminths are parasitic worms that produce a variety of immunomodulatory molecules to regulate host immune responses with a goal to survive within the host. As a core component of innate and adaptive immunity, the complement system exerts direct anti-pathogen effects and enhances the functions of effector immune cells [26,27], playing a crucial role in defending against pathogen invasion and maintaining homeostasis. Many pathogens have evolved sophisticated strategies to circumvent complement attack for their survival. Calreticulin (CRT) is one of the immunomodulatory proteins expressed by the parasitic pathogens targeting host complement C1q such as CRT from *Trypanosoma cruzi* [28], *Trichinella spiralis* [29], and *Brugia malayi* [30].

Our previous findings demonstrated that infection of *E. multilocularis* induced antibodies against calreticulin (*Em*CRT), and that immunization with recombinant *Em*CRT produced a mixed Th1/Th2 immune response in BALB/c mice which elicited partial protection against *E. multilocularis* infection [21], indicating *Em*CRT plays a critical role in the survival of the parasite in the host. Further investigations in our group identified *Em*CRT was a strong immunomodulatory protein which inhibited host complement activation and complement-related immune responses through binding to C1q [8] and mannose-binding lectin (MBL) [31]. In our previous study, we narrowed down the binding region between r*Em*CRT and C1q to the S domain (140–292 aa), which spans the N and P domains [19]. Identification of the precise C1q-binding site within *Em*CRT-S at the amino acid level would facilitate the better understanding of the immunomodulatory mechanism of *Em*CRT involved in regulating complement activation and other immune cells and would also provide key information to design and develop peptide vaccines against echinococcosis and therapeutic drugs for complement-associated inflammatory or autoimmune diseases.

To pinpoint the C1q-binding site in *Em*CRT, in this study we expressed overlapped fragments within *Em*CRT-S. Through binding assay with the expressed recombinant fragments we further narrowed down the binding site to residues 140–204 (r*Em*CRT-S1), which mainly interacts with the C1q A chain (Figure 2). As documented, the C1qA chain plays a key role in recognizing C-reactive protein and associated molecules [32]. To further determine the amino acids within *Em*CRT-S1 that interact with C1q, we designed and synthesized 11 overlapped peptides (15 amino acids, P1–11) that cover the whole sequence of *Em*CRT-S1. Dot-blot assay with the synthesized peptides demonstrated that only the P5 peptide (160–174 aa) bound to C1q (Figure 3), whereas the adjacent overlapping peptides P4 and P6 did not showed any binding activity. Specifically, P4 lacks the 170–174 aa region of P5, while P6 is deficient in the 160–164 aa segment of P5. Neither of these adjacent peptides can present the native epitope in an intact linear conformation, which suggests that the minimal linear epitope recognized by C1q is entirely localized within P5 and most likely concentrated in its central core region. These findings confirm that the C1q-binding site on *Em*CRT is a linear motif strictly restricted to amino acids 160–174 and that overlapping peptide screening is an effective approach to delineate the boundaries of this binding region. Based on the current data, it can be inferred that the minimal C1q-binding epitope resides within P5. To further precisely identify the amino acid residues involved in the *Em*CRT-C1q interaction, shorter peptides within P5 and site-directed mutagenesis will be required. Functional assays with P5 further revealed that the P5 peptide was able to inhibit C1q-initiated classical complement activation demonstrated by the significantly reduced C3b and C4b deposition and hemolytic activity of sensitized erythrocytes at a similar level to that of the full-length *Em*CRT (Figure 4). All results support that the P5 peptide serves as the C1q binding site on *Em*CRT that inhibits the C1q-initiated classical complement activation pathway.

As a pattern recognition molecule, C1q not only initiates the complement activation cascade but also directly interacts with receptors on the surface of various immune cells such as neutrophils, mast cells, macrophages and dendritic cells, thereby regulating cell chemotaxis, activation, and effector functions [8,11,33]. Neutrophils are important effector cells in defending against pathogen infections through phagocytosis, formation of neutrophil extracellular traps (NETs) and release of antimicrobial proteins such as ROS, MPO and Cath-G [34]. In this study, we identified that P5 significantly suppressed the C1q-induced chemotactic migration of neutrophil-like cells (dHL60) (Figure 5) and reduced the production of ROS, MPO and Cath-G in these cells (Figure 6). These findings demonstrate that the P5 peptide effectively impairs C1q-associated activation of multiple immune cell effector functions through binding to C1q and blocking its binding to the C1q receptor on neutrophil cells. In many autoimmune diseases such as rheumatoid arthritis (RA), neutrophil-derived ROS, Cath-G and MPO are critical pathogenic mediators contributing to the disease progress [35]. Excessive ROS production promotes synovial inflammation and disturbs immune homeostasis. In patients with RA, ROS maintain abnormally high levels in the articular synovium and inflammatory microenvironment, leading to persistent oxidative stress. Oxidative stress not only causes damage to biological molecules such as DNA, lipids and proteins, but also aggravates local hypoxia. This further promotes the activation of pro-inflammatory signaling pathways and in turn exacerbates chronic inflammatory responses. In addition, ROS serve as key signaling molecules to regulate the activation and polarization of immune cells, as well as the expression of inflammatory mediators, thereby driving the inflammatory and tissue-destructive pathological processes of RA [36]. Cath-G activated in acidic environments degrades cartilage and bone matrix and promotes neutrophil migration, chemokine processing, and NETs formation. The activity of cathepsin G is significantly elevated in the synovial fluid of patients with RA. As a monocyte chemoattractant, it recruits monocytes to synovial lesions. Moreover, cathepsin G can induce the degradation of articular cartilage at the cartilage–pannus junction [37,38]. MPO catalyzes the production of hypochlorous acid which damages glycosaminoglycans and articular cartilage. Both MPO and HOCl, its primary product, have been assumed to be massively involved in the degradation of the polymeric components (particularly GAGs of the proteoglycans) of cartilage for many years, and characteristic degradation products of cartilage (acetate and oligosaccharides) could be detected in the inflamed joint fluids from patients suffering from RA. Enzymes including cathepsin G and MPO enable neutrophils to efficiently eliminate pathogens ingested during phagocytosis and to contribute to extracellular defense mechanisms such as degranulation and the release of NETs [39]. These components also cause inflammation and immunological damage in autoimmune diseases. The significant inhibition of these inflammation-associated proteins through the binding of P5 to C1q indicates P5 could be a counterpart to C1q-related inflammation.

Identification of the C1q-binding peptide in *Em*CRT provides the potential therapeutic drug target to treat complement-related inflammatory and autoimmune diseases. Up until today, the development of such drugs remains limited with a few complement-targeting agents in clinical trials mainly due to specificity, safety, and long-term efficacy concerns [40,41]. Identification of the precise C1q-binding site in *Em*CRT at the amino acid level would not only illuminate the immunomodulatory mechanism of *Em*CRT but would also facilitate rational drug design. Except for the therapeutic target for inflammatory and autoimmune diseases based on its effective binding ability to C1q, the P5 peptide could also be a potential vaccine target for echinococcosis due to its key role in evading host C1q-initiated complement attack. Immunization of the parental r*Em*CRT elicited significant protection against *E. multilocularis* infection in mice [21]. Due to its easy manufacturing, less regulatory concern and precise targeting on the antigen functional epitopes, peptide vaccines have become a novel approach to developing vaccines against infections or cancer therapy [42,43,44]. The development of monoclonal antibodies targeting the functional peptides or domains also acts as a tool for the treatment of infectious diseases. A monoclonal antibody targeting the complement C9-binding domain of *Trichinella spiralis* paramyosin (Ts-Pmy) has been successfully generated with strong inhibition on the complement-binding activity of Ts-Pmy, leading to a marked enhancement of complement-dependent killing of newborn larvae in vitro and decreased infectivity of *T. spiralis* larvae in passive antibody-transferred mice [45,46,47].

## 5. Conclusions

In this study, we successfully pinpoint the C1q-binding site to 15 amino acids (P5) within *Em*CRT (160–174 aa) that bind to C1q and inhibit C1q-intiated classical complement activation and C1q-associated neutrophil activities. This finding provides a novel approach to developing vaccines against echinococcosis or therapeutic drugs for inflammatory or autoimmune diseases using a functional peptide that is easily manufactured with lower costs and less regulatory or safety concerns. The potential of C1q-binding P5 peptide as a vaccine or therapeutic drug is under investigation.

## Data Availability

Data included in this article is available from the corresponding author upon request.
